# Molecular mechanisms underlying noncoding risk variations in psychiatric genetic studies

**DOI:** 10.1038/mp.2016.241

**Published:** 2017-01-03

**Authors:** X Xiao, H Chang, M Li

**Affiliations:** 1 Key Laboratory of Animal Models and Human Disease Mechanisms of The Chinese Academy of Sciences and Yunnan Province, Kunming Institute of Zoology, Kunming, Yunnan, China

## Abstract

Recent large-scale genetic approaches such as genome-wide association studies have allowed the identification of common genetic variations that contribute to risk architectures of psychiatric disorders. However, most of these susceptibility variants are located in noncoding genomic regions that usually span multiple genes. As a result, pinpointing the precise variant(s) and biological mechanisms accounting for the risk remains challenging. By reviewing recent progresses in genetics, functional genomics and neurobiology of psychiatric disorders, as well as gene expression analyses of brain tissues, here we propose a roadmap to characterize the roles of noncoding risk loci in the pathogenesis of psychiatric illnesses (that is, identifying the underlying molecular mechanisms explaining the genetic risk conferred by those genomic loci, and recognizing putative functional causative variants). This roadmap involves integration of transcriptomic data, epidemiological and bioinformatic methods, as well as *in vitro* and *in vivo* experimental approaches. These tools will promote the translation of genetic discoveries to physiological mechanisms, and ultimately guide the development of preventive, therapeutic and prognostic measures for psychiatric disorders.

## Recent genetic analyses of neuropsychiatric disorders

Schizophrenia, bipolar disorder, major depressive disorder and autism are highly prevalent complex neuropsychiatric diseases across the world populations with varied clinical symptomatology.^1^ The underlying etiology of these neuropsychiatric disorders remains largely unknown, but a number of family, twin and adoption studies have revealed moderate-to-strong genetic elements in these illnesses.^2, 3^ Although substantial heritability of neuropsychiatric disorders suggests that genetic approaches may provide valuable information about its biology, early linkage and association analyses in small samples yielded limited success because of the phenotypic heterogeneity and genetic architecture complexity in most psychiatric conditions. Recent large-scale genome-wide association studies (GWASs) through international collaborations suggest that these psychiatric disorders, though all featured genetic heterogeneity,^4^ share varying degrees of overlap in genetic risk components.^5^ These studies have significantly boosted our knowledge of reliable genetic variants associated with the diseases.^6, 7, 8, 9, 10^

Some examples of these GWASs include the Psychiatric Genomics Consortium (PGC2) GWAS of schizophrenia^8^ as well as Converge consortium GWAS and 23andMe GWAS of major depressive disorder.^6, 10^ The PGC2 GWAS, which identified 108 independent associated loci for schizophrenia,^8^ is of epoch-making significance. Most of these identified risk variants shed great light on the pathogenesis of psychiatric illnesses. In the PGC2 schizophrenia GWAS,^8^ 75% of the 108 independent risk loci contained protein-coding genes (40% include one single gene), and a further 8% were within 20 kb of a gene. In fact, many genes within those GWAS risk loci were found to play important roles in neurodevelopment and brain function. For example, *GRM3*, *GRIN2A*, *SRR* and *GRIA1* were known to involve in the neurotransmission mediated by glutamate signaling and synaptic plasticity. Some risk loci also contained genes encoding voltage-gated calcium channel subunits (*CACNA1C*, *CACNB2* and *CACNA1I*) that play pivotal physiological roles. Indeed, genes involved in glutamate signaling, synaptic plasticity and calcium channel activity were also previously implicated in rare genetic variation studies of schizophrenia.^11, 12, 13^ Moreover, the genes relevant to the major hypotheses for schizophrenia pathogenesis (for example, *DRD2*, a validated target for antipsychotic drugs) have also been confirmed in the GWASs,^8^ adding further evidence for these hypotheses. Taken together, the convergence at a broad functional level between studies of common and rare genetic variations suggests that the current genetic approaches are normally reliable.

In addition to its reliability, GWAS is also highly efficient for genetic risk factor identification. It is capable of capturing not only single risk loci^7, 8, 10^ but also polygenic components in psychiatric diseases. These polygenic components usually involve thousands of common alleles of small effects.^14^ They are particularly meaningful when determined simultaneously in one study, in which case they could represent biological pathways associated with disease risk. Taking the recent PGC2 schizophrenia GWAS, for example,^8^ the defined risk loci supported the neuropsychopharmacologic hypotheses that both dopamine D2 receptor and *N*-methyl-D-aspartate receptor were involved in schizophrenia pathogenesis. Similarly, another pathway analyses based on available GWAS data suggested that malfunction of pathways in histone methylation, neuroimmunology and synaptic signaling transmission likely contributed to the etiology of psychiatric disorders.^15^ As such, new therapeutic hypotheses can be proposed and ultimately tested based on the underlying molecular mechanisms of genetic risk associations revealed by GWAS.^16^

Although generally accepted by the scientific community for the reliability and efficiency, the GWAS results are sometimes under debate for the normally small effect sizes of the identified risk loci. This is probably because of the fact that the allele frequencies in patients and controls typically differ by only <2% (the difference between odds ratio is <1.1),^6, 7, 8, 9, 10, 17, 18^ whereas such variants still yield strong statistical association in GWAS analyses. However, the small effects of single locus at the individual subject level do not mean that the biological implications are necessarily trivial. One example is *DRD2*, the gene encoding the dopamine D2 receptor. The GWAS variant at this locus was shown to increase the risk of schizophrenia by <10% with an odds ratio of 1.08 at the population level,^8^ whereas the dopamine D2 receptor has been a primary target of most antipsychotic interventions.^19^ Therefore, the effect size of a given DNA variant on genetic risk for psychiatric disorders does not always reflect the therapeutic value of its affected molecule or biological pathway, and the GWAS-identified risk loci could provide valuable clues. However, whether a certain GWAS locus is translatable into clinical treatment for psychiatric illnesses still largely depends on the relevance of the proteins and/or transcripts affected by susceptibility genes to common biological pathways.

The idea of translating the bulk of GWAS data to clinical application is compelling, but the actual implementation is not always easy. The vast majority of GWAS risk regions contain numerous high linkage disequilibrium noncoding variants with unknown function, and span wide genomic area of multiple candidate genes.^8^ For this reason, identifying the specific gene(s) accounting for the clinical association remains a hassle. Considering these characteristics of the current enormous GWAS results, several following important questions should be first asked when utilizing such genetic data to uncover the relevant disease biology. (1) Are there any specific pathogenic gene or transcription products in the particular loci explaining the genetic risk association? (2) Are there any causal variant(s) in certain genomic regions responsible for the disease risk? (3) What are the mechanisms through which the causal variant(s) affect gene expression/function? Moreover, (4) are there physiological consequences of the genetic risk factors? If so, are they related to disease-associated pathological alternations?

**Figure 1:**
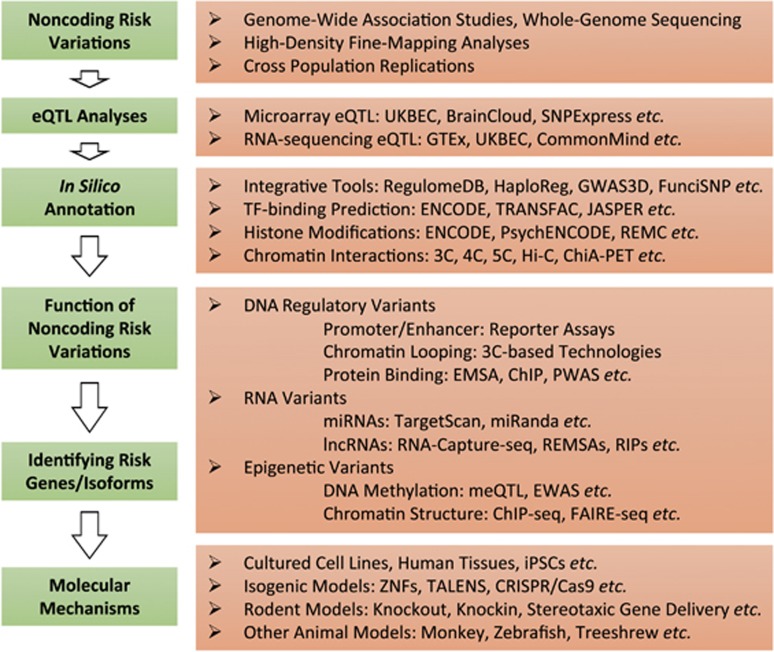
Workflow for functionally analyzing and interpreting noncoding risk loci. 3C, chromosome conformation capture; 4C, circular 3C; 5C, carbon-copy 3C; eQTL, expression quantitative trait locus.

Taken together, GWAS provides valuable information for psychiatric disease research, but efforts are required to tackle possible problems. Here, we have reviewed recent progress in functional evaluation of noncoding variations in psychiatric genetic studies (including GWAS and candidate gene analyses), and have proposed a stepwise pipeline to follow the risk loci identification for prioritizing putative functional variations (Figure 1). Through integrating genomic data, computational approaches and biological assays, we sought to elucidate the function of noncoding GWAS risk loci and their roles in psychiatric illnesses.

## Translating genetic susceptibility loci to risk molecules

### Utilization of brain eQTL databases

To understand the roles of noncoding risk loci in disease progression, it is important to predict their functional and physiological impact on human health. Among the numerous risk variants defined by genetic analyses such as GWAS, exome-sequencing or candidate gene studies, nonsynonymous variants are perhaps the easiest to illustrate for the manifestation of protein structure or function alternations.^20^ Indeed, missense variants in specific risk genes have been found to significantly associate with psychiatric disorders in both GWAS and whole-exome sequencing studies (for example, rare loss-of-function variants in *SETD1A*).^13, 20, 21, 22^ However, as discussed above, most psychiatric risk loci are located in noncoding genomic regions with unknown functionality.^8^ This is probably explained by the accumulating consensus that genetic architecture of psychiatric disorders involves sequence variations that primarily play a role in gene regulation or processing rather than sequences of encoded proteins.^23^ Therefore, it is necessary to use transcriptomics data combined with genotyping in neural tissues to identify genes or transcripts associated with risk genotypes. These genes and/or transcription products are considered ‘true signals' that are critical in linking the information from genetic associations to functional consequences, mostly through their expression quantitative trait locus (eQTL) effects.

As stated by the name, eQTL provides hints for transcriptome (for example, promoter regulation, enhancer, splicing, microRNA, long noncoding RNA and epigenetic processes) alterations attributed to specific genetic risk factors. As we believe that psychiatric risk variants that do not result in protein coding changes usually manifest as an effect on transcriptomic outcomes, integrative analyses of GWAS and eQTL data are crucial in understanding genetic mechanisms and discovering targetable molecules. In fact, several recent studies have achieved great success following this strategy. For example, the major histocompatibility complex region on human chromosome 6 spanned over hundreds of genes and contained numerous variants conferring risk of schizophrenia in PGC2 GWAS,^8^ but little was known about the underlying molecular mechanisms of this genomic region. Sekar *et al.*^24^ recently identified that the structurally diverse alleles of the *complement component 4* (*C4*) genes were the major contributors. They reported that those alleles caused widely varying levels of *C4A* and *C4B* expression in the brain, and the association of common *C4* allele with schizophrenia was proportional to its tendency to increase the expression of *C4A*.^24^ They further revealed localization of human *C4* protein in the neuronal synapses, dendrites, axons and cell bodies, as well as important roles of murine *C4* in synapse elimination during postnatal development.^24^ Overall, this study suggested that excessive dosage of *C4* led to increased postnatal synaptic pruning, providing one of the potential mechanisms for the previously observed gray matter loss^25^ and synaptic structure impairment in schizophrenic brains.^26^ They illustrated an excellent model for the meticulous identification and characterization of causal, small effect size, common loci found in GWAS. Another interesting example is the series of studies on *ANK3* in bipolar disorder. Genetic loci spanning the *ANK3* gene were previously reported in several bipolar disorder GWASs.^7, 27, 28, 29^ A recent study found a loss-of-function variant in a minor isoform of *ANK3* that disabled the proper splicing of the protein, resulting in protection against bipolar disorder.^30^ Intriguingly, another study also described that allelic variation at the bipolar disorder risk single-nucleotide polymorphism (SNP) was correlated with a significant difference in cerebellar expression of a brain-specific *ANK3* transcript.^31^ Although these studies reported different SNPs at *ANK3* (and in very low linkage disequilibrium, *r*^2^=0.001),^30, 31^ they together suggested a *cis*-regulatory transcriptional effect of *ANK3* that was relevant to bipolar disorder pathophysiology.

The eQTL data are both informative and easily accessible. There have been a number of eQTL databases with varying sample sizes generated from certain groups (Table 1). These databases present valuable brain transcriptomics (microarray and RNA sequencing) data in post-mortem tissues that has long been an essential substrate for investigating the molecular pathology of psychiatric disorders.^32, 33^ To date, the major consortia generating brain eQTLs are the GTEx (Genotype-Tissue Expression), the United Kingdom Brain Expression Consortium (UKBEC) and the CommonMind consortium. The original eQTLs in GTEx and UKBEC were derived primarily from microarray technology, but have now switched over to RNA-sequencing approach. The initial version of GTEx (http://www.ncbi.nlm.nih.gov/gtex/GTEX2/gtex.cgi) contained microarray data from studies on human brain cerebellum, frontal cortex, caudal pons and temporal cortex (*N*=150).^34^ The newer GTEx Project data are RNA-sequencing results from a variety of human brain regions and are available through the GTEx Portal (http://www.gtexportal.org/home/).^35, 36^ UKBEC presented microarray eQTL data from 10 human brain regions (www.braineac.org) (*N*=134).^37, 38^ They have also generated RNA-sequencing data on substantia nigra and putamen in post-mortem control brains (*N*=65 and 105, respectively). The CommonMind consortium (http://commonmind.org/WP/) generated data modalities (RNA and DNA sequencing, genotyping) across multiple brain regions (dorsolateral prefrontal cortex (DLPFC), anterior cingulate cortex and superior temporal gyrus) from schizophrenia, bipolar disorder and control samples, totaling a collection of >1000 samples. The CommonMind consortium started the first data release of SNP genotypes and RNA-sequencing results for the DLPFC of over 600 subjects registered at the Mount Sinai, University of Pittsburgh and University of Pennsylvania brain banks since March 2015. Recently, they have published RNA-sequencing analyses of DLPFC from people with schizophrenia (*N*=258) and controls (*N*=279), and have reported several schizophrenia-associated eQTL genes.^39^ Besides, Lieber Institute has published a genome-wide microarray eQTL analysis in human DLPFC of healthy controls (that is, BrainCloud, *N*=269).^32^ They also generated RNA-sequencing data with DLPFC and other brain tissues in an expanded sample size including psychiatric patients and healthy individuals, and plan to publically release the RNA-sequencing results upon publication.

### Cautions needed during eQTL analyses

Although eQTL data provide essential information, cautions are needed when using these expression data sets. First, the eQTLs should be considered ‘authentic' only if they could be replicated across independent samples. A prior report revealed low-to-moderate overlap between eQTL loci across earlier microarray-based eQTL studies^32, 34, 40, 41, 42, 43, 44^ (the percentage of overlapped eQTL is from 0 to ∼35.4% between pairwise brain studies, as shown in Table 4 of the study by McKenzie *et al.*^45^) that might be explained by the different analytical methodologies applied in each study. For example, in the study of Myers *et al.*,^42^ the analyses were based on pooled expression data from three different cortical regions (frontal, temporal and parietal) with uncontrolled covariates, such as brain pH value and microarray batch effects. Only a limited number of the significant *cis*-associations observed in their study survived multiple corrections.^42^ In parallel, Liu *et al.*^41^ performed a brain eQTL analysis focusing on the prefrontal cortex with a statistical procedure optimized for possible confounders. They used surrogate variable analysis^46^ to adjust for covariate effects and ComBat^47^ to minimize microarray batch effects before the eQTL analyses. This procedure improved the detection power by removing sources of nongenetic variations from the data, and they have identified an exceedingly large amount of *cis*-eQTL associations that could stand the strict statistical corrections for multiple testing.^41^ As such, appropriate analytical methods are obviously pivotal in the eQTL analyses, and methodology improvement are always needed. In line with this idea, CommonMind consortium adjusted for known (for example, RNA integrity, library batch, institution (brain bank), age at death, genetic ancestry, post-mortem interval and sex) and hidden variables detected by surrogate variable analysis (conditional on diagnosis but excluding ancestry) in a recent DLPFC RNA-sequencing eQTL analysis of gene expression in European-ancestry subjects.^39^ The adjusted expression then underwent eQTL analyses against genotypes, covarying for ancestry and diagnosis. A comparison of the identified eQTLs in the CommonMind study^39^ with previously reported DLPFC eQTLs^32, 34, 36, 38, 48^ showed that the CommonMind sample not only captured most eQTLs found in other independent samples, but also discovered a substantial number of genes with previously undetected eQTLs (details shown in Table 1 of the CommonMind study^39^).

Second, in-depth mRNA characterization are necessary to identify potentially pathogenic transcripts during eQTL analyses. It is well known that multiple mRNA isoforms may arise from one single gene but differ in their expression levels and/or functions in cells or organs. In some cases, psychiatric risk variants may affect only specific transcripts of a given gene, such as *AS3MT*, *ZNF804A*, *KCNH2* and *NRG1*.^49, 50, 51, 52^ Li *et al.*^51^ have previously reported a novel truncated *AS3MT* isoform (*AS3MT*^d2d3^) lacking two exons compared with the full-length protein. The *AS3MT*^d2d3^ was strongly associated with schizophrenia risk SNPs in the 10q24.32 genomic region. The fact that only *AS3MT*^d2d3^ but not the full-length *AS3MT* was brain enriched, human specific and upregulated during early neuronal differentiation suggested divergent roles between them. Another example is the discovery of a novel truncated *ZNF804A* splicing variant.^52^ *ZNF804A* was previously reported to contain a genome-wide significant risk variant rs1344706 for schizophrenia.^53, 54, 55, 56^ Researchers later found that such risk variants might contribute to schizophrenia pathology via altered expression of *ZNF804A*, as significant associations of rs1344706 with *ZNF804A* gene expression levels was observed in fetal brain samples.^57, 58, 59^ Taken one step further, Tao *et al.*^52^ discovered a novel *ZNF804A* isoform with an alternative 5′ untranslated region and translation start site using a 5′ RACE (rapid amplification of cDNA ends) assay. This novel truncated *ZNF804A* isoform, rather than the full-length *ZNF804A* transcript, was associated with rs1344706 in fetal samples.^52^ These data suggested that the association between rs1344706 and *ZNF804A* gene expression might originate from the generation of this novel truncated isoform. Both *AS3MT* and *ZNF804A* stories indicate that RNA splicing plays an important role in psychiatric diseases. In fact, RNA alternative splicing has been proposed to be a primary mechanism for genetic variation in disease progression, and many GWAS signals for common traits involved alternative splicing.^60^ Regarding this, characterizing the transcript structures of a risk gene is pivotal for our general understanding of the disease biology. To study this, the current human mRNA data sets (Ensembl and UCSC) are handy tools. Normally, the diverse transcript structures of a gene can be retrieved in these data sets. However, in the event that novel transcripts exist for a defined gene, one will need to identify them via RNA-sequencing analyses. Specifically, junction-level analyses in RNA-sequencing data sets should be carried out. Junctions are the RNA-sequencing read counts spanning at least two exons, and junction reads between nonadjacent exons (that is, exon-skipping junctions) indicate alternative splicing. Following RNA-sequencing analyses, the transcripts of interest are further investigated using experiments such as RACE and end-to-end PCR. This strategy will provide clues for pathogenic gene/protein product that is vital for future model building based on the biological mechanisms, and development of relevant drugs and therapies.^61^

Another important issue to consider during eQTL analyses is the temporal and spatial conditions that interact with the identified molecular mechanisms. In a biological system, certain regulatory effects might exist only in some cell types and/or at particular developmental stages, or even under certain biological conditions. Given that some eQTLs and most disease manifestations are tissue specific,^38, 62^ it is recommended that eQTL analyses be performed in the disease-relevant tissues. For example, eQTLs from adipose tissue have shed great light on obesity-related risk loci,^63^ and eQTLs from lymphoblastoid cell lines have helped explain genetic risk loci for immunological diseases.^64^ This idea has also been applied in research for the schizophrenia risk variation at the *ZNF804A* locus. The genome-wide significant SNP rs1344706 associated with the expression of a truncated *ZNF804A* isoform at the early stage of human fetal brain development.^52^ It was therefore important to establish where and when psychiatric risk variants exerted their effects. This easiest way to obtain such information is through publicly available databases. For example, the BrainCloud provides valuable resources of transcriptome expression data from post-mortem DLPFC of normal human subjects across the lifespan (that is, from fetal development through aging). It is thus possible to stratify the subjects based on the developmental stages (for example, prenatal and postnatal) and examine the role of the DNA variant at relevant ages.^32^ Meanwhile, the UKBEC consortium has produced mRNA expression data for 10 human brain regions from control individuals, establishing a comprehensive data set for regional specificity of gene expression regulation across human brain.^38^

Next, the varying sample sizes of brain eQTL databases can potentially affect the reliability of eQTL analyses. It is known that the power to detect eQTL is partly a function of sample size. Although it was estimated that a sample size of 100 individuals is sufficient for 80% power in eQTL studies,^65^ the power to detect an effect across multiple studies is reduced when an expression SNP has a subtle effect size or when multiple genomic loci simultaneously control transcript expression levels. On the other hand, the effect size of expression SNP on gene expression has substantial influence over the power of detecting an eQTL association. For example, Wang *et al.*^66^ previously showed that when the eQTL effect size increased from 0.5 phenotypic s.d. (σ) to 4σ, the power of detecting a significant association (*P*<0.05) increased from 10.5 to 100% (Table 5 in Wang *et al.*^66^ study), and the bias of observing authentic effect also decreased (Figure 10 in Wang *et al.*^66^ study). As such, larger sample sizes and the expression SNP effect sizes should increase the statistical power for eQTL effect detection.

Last but not least, potential limitations of eQTL analyses should be considered. First, the majority of identified eQTLs are considered to be *cis*-acting and arbitrarily defined to regulate genes within 1 Mb on a chromosome.^67^ However, genetic variants can also affect the expression of genes residing further away or even on different chromosomes (defined as *trans*-eQTLs, although they incur a greater penalty for multiple testing, require greater power for detection and are more prone to false positives that are less replicable than *cis*-eQTL).^68^ For example, Fehrmann *et al.*^69^ reported independent *trans*-associated SNPs that affected similar genes, suggesting that independent GWAS associations might influence similar biological pathways. Second, the impact of haplotypes on eQTL effects should be factored in.^70^ As linkage disequilibrium patterns are usually population specific, associations between variants that tag a haplotype could lead to ambiguous identification of the true casual variant. Third, the targets of eQTL associations could be either coding or noncoding RNAs,^71^ whereas the latter still remains to be explored. Finally, the mapping of regulatory variants for any complex trait within single or certain populations are often less accurate because of various factors, such as genotyping issues.^72, 73, 74^ These limitations should be always acknowledged, and eQTL analyses for complex illnesses/conditions should always be conducted across diverse populations.

Collectively, with the help of eQTL analyses, it is possible to identify a molecular mechanism in the brain transcriptome that accounts for the genetic association detected in clinical samples. Such results will guide the subsequent *in vitro* and *in vivo* testing for the identified molecules/pathways.^61^

## Differential expression between psychiatric patients and controls

Alongside eQTL analyses, genes that are differentially expressed between patients and healthy controls may play key roles in the pathogenesis of psychiatric disorders and aid in the identification of molecular mechanisms underlying genetic risk loci. That is to say, if a gene (or a transcript) shows strong eQTL association with psychiatric genetic risk loci as well as the illness state, and the risk-associated genotype predicts the same direction of expression difference between cases and controls, it would be an ideal target for further functional studies. Indeed, recent advances in microarray or RNA sequencing techniques have allowed researchers to dissect the roles of gene expression alterations in the pathogenesis of psychiatric disorders including bipolar disorder,^75, 76, 77, 78, 79, 80, 81, 82^ major depressive disorder^83, 84, 85^ and schizophrenia.^79, 80, 86, 87, 88, 89, 90^

However, only a small number of genome-wide significant differentially expressed genes have been reported so far, and only few overlapping differentially expressed genes were reproduced across different studies (for the same illness). This is likely resulted from the multiple confounding factors such as relatively small samples sizes, sample heterogeneity and other technical reasons including instability of RNA and the post-mortem conditions (for example, brain tissue pH changes, coma, respiratory arrest, hypoxia, seizures, dehydration, multiple organ failure and head injury). These confounders may interfere with the relationship between measured gene expression levels and disease status^91^ to cause difficulty in capturing the desired signals. The following paragraphs will discuss these problems in details.

Among all possible confounding factors, sample size is undoubtedly one major issue that is very difficult to resolve because of the nature of the affected organ in psychiatric disorders. In the past, people applied stringent multiple corrections in genome-wide expression analyses, resulting in identification of extremely significant effects at the cost of substantial false negatives. Such problem becomes harder to resolve in the current gene expression studies that have typically used RNA samples from either brain tissues or peripheral blood.^92^ Obviously, brain is considered to be the most relevant tissue as its dysfunctions are presumed to be the origin of psychiatric illnesses. However, the brain tissues can only be collected after the participant is deceased, and this significantly affected the total sample size, let alone the number of samples for cases. In fact, this disadvantage is reflected in many of the current brain eQTL resources, in which only healthy controls are utilized. Blood has been an alternative tissue for eQTL analysis. Although it may appear less direct for understanding psychiatric disorders, it is relatively easy to collect. So far, it is generally accepted that blood RNA analyses do provide some clues for the understanding of psychiatric disorders.^84, 90^

Besides the problems with sample size, technical limitations exist in the current differential expression analytic methods. Microarray analyses with a single probe (or several probes) per gene usually lack ideal resolutions for transcripts. Similarly, insufficient sequencing depth in RNA-sequencing analyses may preclude low-abundant transcripts that are important in the pathogenesis of psychiatric disorders from detection. Taken together, further differential expression studies comparing cases and controls involving larger brain sample sizes and more in-depth RNA-sequencing are necessary.

## Resources and bioinformatics analyses to predict regulatory variants

After obtaining the evidence for a link between DNA genotype and gene regulation or processing through eQTL analyses and differential expression analysis, a range of further molecular approaches are required to elucidate the regulatory mechanisms. Public resources and bioinformatics analyses are useful and easily accessible tools that usually provide data suggesting multiple functional component in the genome.

The most well-known and representative bioinformatics resources are the data sets of regulatory elements in noncoding DNA regions obtained through high-throughput sequencing techniques. These regulatory DNA sequences are characterized by an open chromatin for the access of transcription factors (TFs) that modulate gene expression. Specifically, during the transcription of a gene, RNA polymerase II, numerous TFs and accessory molecules are recruited to the gene promoter region. Through binding to the regulatory sequences, transcription initiation complex is formed to start the basal transcription machinery. In this process, the ease of these molecules to access the DNA, which is regulated by posttranslational histone modifications (methylation, acetylation and so on), is the ultimate determinant. Previous studies suggested that a proportion of psychiatric risk variants located within the regulatory sequences (such as promoters and enhancers) influenced gene expression through transcriptional, posttranscriptional and posttranslational (for example, posttranslational modifications of histones or RNA polymerase II as regulation of transcription) mechanisms.^93, 94, 95^ Importantly, regulatory signals can act over long genomic distances when brought into contact with target promoters by three-dimensional DNA folding. To reveal such mechanisms conveyed by specific genomic variants, computational prediction can be conducted based on recent established large-scale genome-wide data sets (Table 2) such as the Encyclopedia of DNA Elements (ENCODE)^96^ and the Roadmap Epigenomics Mapping Consortium (REMC).^97^ These data sets can be routinely mined with multiple computational tools (for example, RegulomeDB,^98^ HaploReg,^99^ GWAVA^100^ and FunciSNP^101^) for the annotation of DNA variants.

Initiated as a follow-up to the Human Genome Project (Genomic Research), ENCODE project aimed to map the functional elements in the genome, usually defined as a segment of the genome having either a biochemical signature (for example, TF-binding site or some other protein-binding site) or a specific chromatin structure (for example, accessible open chromatin), and encodes a product (for example, a protein).^96^ The initial phase data are available through the UCSC (University of California, Santa Cruz) Genome Browser,^102^ Table Browser tool^103^ and the FTP site (ftp://hgdownload.cse.ucsc.edu/goldenPath/hg19/database/). The most recent releases are available from the ENCODE website (https://www.encodeproject.org/). The ENCODE consortium has generated data from both cell lines and human brain tissues. For cell lines, they categorized multiple cell lines into three tiers based on the respective superiority for biological experiments. For example, tier 1 cell lines such as GM12878, H1-hESC and K562 have the highest priority with regard to designing experiments. They later included two cancerous brain cell lines in its 2012 version: glioblastoma (Gliobla) and neuroblastoma (SK-N-SH, and sublines SH-SY5Y and retinoic acid-treated SK-N-SH_RA; also SK-N-BE and clone BE2_C). Though neither of these were in tier 1, they were valuable addition to the database given their nature as brain cells. One major limitation though is that data generated using immortal cell lines may not represent the actual biology in normal cells and tissues. This concern was later addressed when ENCODE added new data from various sources including human brain tissues since the 2012 release. For instance, processed DNase I hypersensitivity data ‘peaks' (regions of statistically significant enrichment based on the signal from the measurement of background abundance in the genome) were available from tissues of nine human brain regions (frontal cortex, cerebellar cortex, cerebellum, globus pallidus, midbrain and middle frontal gyrus), two fetal brains and five primary brain cell types (astrocyte of the cerebellum, astrocyte of the hippocampus, brain microvascular endothelial cell, brain pericyte and choroid plexus epithelial cell). Generally, ENCODE is the first large international collaborative project mapping functional elements in the genome. Its standardized and robust data have greatly contributed to our roadmap from functional annotation prediction to laboratory testing.

Besides mapping the functional elements in the genome, available data sets for epigenome provide another layer of information for gene expression regulation. One major form of epigenetic modification is DNA methylation that involves the enzymatic addition of a methyl group to the carbon-five position on cytosine residues.^104^ Currently, data from brain tissue are available for allele-skewed methylation (also referred to as methylation quantitative trait loci (meQTL) among other terms), a scenario in which one allele shows significantly different methylation levels compared with another allele at the same base pair location.^105, 106^ Intriguingly, in two recent genome-wide brain DNA meQTL analyses,^107, 108^ enrichment of meQTL signals among risk loci in schizophrenia GWAS^8^ was observed. These data suggested that meQTL might be useful in refining GWAS loci via analyzing discrete sites of DNA methylation in the brain that are associated with schizophrenia (and other psychiatric) risk variants.^109^

In addition to DNA methylation on cytosine bases, other forms of epigenetic DNA modifications (for example, hydroxymethylation) have been recently reported and require further analyses.^110^ Meanwhile, mechanisms other than DNA methylation (for example, histone modifications and DNase I hypersensitivity) can also regulate DNA accessibility and gene expression.^111^ Mapping the epigenome with these information are also potentially helpful for understanding the brain biology and psychiatric illnesses. The NIH REMC^97^ (http://www.roadmapepigenomics.org/) has undertaken this task and mapped the DNA methylation, DNA accessibility and RNA expression in primary human tissues. Although the sample sizes are tiny (usually one or two samples), there are histone modification and RNA-sequencing data from up to eight adult brain regions (hippocampus middle, substantia nigra, anterior caudate, cingulate gyrus, interior temporal lobe, angular gyrus and dorsolateral prefrontal cortex) and fetal brains. They also possess data of DNA accessibility mediated by DNase I hypersensitivity in fetal brain. Along with human brains, REMC assessed functional elements in stem cells and primary *ex vivo* tissues. Although such cells are not ideal for epigenome investigation in a living system given the stochastic random epigenetic changes appeared as stem cells divide,^112^ these data have still provided valuable information.

In summary, these programs have tested the potential impact of DNA sequence variation on several genomic features including histone modifications, chromatin immunoprecipitation followed by next-generation sequencing (ChIP-Seq), DNase I hypersensitive sites, chromatin interactions, evolutionary sequence conservation and TF-binding motifs (measured with targeted biochemical assays and high-throughput sequencing technologies). Mapping these data to a genomic region of interest facilitates the design of further functional assays.

Despite the rapid progress in bioinformatic analyses, many of these computational tools, however, are still not exhaustive, and only limited TFs and cell types have been assayed. Moreover, these programs do not take into account tissue specificity, in which case regulatory variations might influence signals in irrelevant cell types. As a result, the high false negative probability (for example, missing data might lead to the absence of valid results) remains a major challenge. To address this, the PsychENCODE project was found^113^ to produce a public resource of multidimensional genomic data using samples from ∼1000 healthy and psychiatric disease-affected human post-mortem brains. The key goal of the PsychENCODE project was to look at regulatory elements (for example, TF-binding sites) as was done by the ENCODE Project, but in post-mortem control and psychiatric (schizophrenia, bipolar disorder and autism spectrum disorder) brains. This project is expected to provide an enhanced framework of regulatory genomic elements, to catalog epigenetic modifications and to quantify coding and noncoding RNA and protein expression. The first release made available a few histone modifications and RNA-sequencing data from individuals or from induced pluripotent stem cell (iPSC)-derived neurons on the PsychENCODE Knowledge Portal (https://www.synapse.org/#!Synapse:syn4921369/wiki/235539). Overall, as the knowledge evolves, projects with consideration of specific biological questions are being launched and present useful preliminary reference for designing functional assays.

Collectively, identifying the functional causal variant(s) among psychiatric noncoding risk-associated loci with public bioinformatics tools is a pivotal step to guide functional assays. Regarding potential advantages and limitations discussed above, information in the following aspects should be synthesized: the strength of clinical risk associations, the eQTL effects on gene/isoform expression, the likelihood that the variant perturbs *cis*-regulatory elements, the relative impact of the variant on reporter gene activity and the evolutionary conservation of the putative regulatory element. Although it is difficult to confidently pinpoint the functional variants underpinning a risk signal, such approaches will prioritize certain variants of interest at associated loci. In addition, using these data to test whether psychiatric risk signals are statistically enriched in regulatory regions of particular cell types provides the information about where these variants are active, and implying relevant biological mechanisms.

## Validations of regulatory effects via *in vitro* functional assays

The eQTL and bioinformatic analyses provide useful tools to predict targeted molecule(s) of psychiatric risk variants. However, this strategy usually only achieves indirect evidence of a molecular association, and experimental testing is necessary to confirm the mechanistic relevance. Thus, we propose that validation of regulatory effects *in vitro* should be the next step. In recent years, there have been considerable advances in useful techniques for studying noncoding genomic loci and uncovering causal variant(s).

As previously discussed, psychiatric risk variants are enriched in *cis*-acting eQTLs in brain.^93, 94, 95^ Underlining the principle that regulatory variations not always affect spatially the closest gene(s), there has been a growing consensus that chromosomal regions frequently fold in order to bring distant regulatory regions in closer proximity to the genes they regulate, such as transcriptional enhancers. These DNA elements are typically located more than 1 kb away from their target genes and regulate transcription through long-range interactions enabled by the formation of chromatin loops.^114^ The recent PGC2 GWAS has screened the credible risk variants within the 108 genome-wide significant schizophrenia loci in 56 human cell lines and tissues, and found significant enrichment in active enhancers in human brain.^8^

To verify these long-range chromosomal interactions, chromatin conformation capture (3C) technique is one of the most reliable experimental approaches. The 3C-based techniques involve first formaldehyde crosslinking of interacting sites in cells of interest, and then cutting of DNA with a restriction enzyme and a ligation reaction to join crosslinked DNA fragments to investigate chromosomal interactions at specific candidate loci.^115^ The 3C-based methods have been utilized in the functional characterizations of GWAS risk-associated loci for several complex traits or diseases such as pigmentation and cancer.^116, 117, 118^ There have also been several studies using 3C-based methods to interrogate psychiatric risk loci.^119, 120^ Roussos *et al.*^120^ used 3C to elucidate the regulatory roles of a psychiatric risk variant in the intron of *CACNA1C*. The risk variant was predicted to locate in an enhancer region that interacted with the *CACNA1C* promoter in human DLPFC and neurons derived from human iPSCs. Using a reporter gene assay, they showed that the risk allele within this enhancer caused lower transcriptional activity, consistent with its association with decreased *CACNA1C* expression in human cerebellum.^121^ Although such result seemed to be in the opposite direction from previous studies that showed that the risk allele predicted higher *CACNA1C* expression in human DLPFC and induced human neurons, such conflict could be because of differential roles of *CACNA1C* in different brain regions.^122, 123^ The knowledge of *CACNA1C* risk variant was further improved by Eckart *et al.*,^124^ who showed that risk variants at *CACNA1C* marked eQTL in the superior temporal gyrus region, and found one SNP rs4765905 showing allele-dependent regulatory function in reporter assays and protein microarrays. Using circular 3C (4C), they revealed interactions of the disease-associated regions (covering the risk SNPs) with *CACNA1C* promoter. In another study of the psychiatric risk gene *MIR137*, Duan *et al.*^119^ identified a rare enhancer SNP near this gene that conferred risk of schizophrenia and bipolar disorder. The risk allele reduced enhancer activity of its flanking sequence by >50% in human neuroblastoma cells, predicting lower expression of *MIR137/MIR2682* that was then also proved with 3C assays (Table 3).

Though being a powerful method for testing chromosomal interaction at specific loci, 3C also has two principle limitations. First, it is unable to distinguish relevant nearby chromatin interactions (within ~20 kb) from background interactions caused by random collisions. Second, 3C can only detect specific interactions between prespecified regions as it relies on PCR primers designed across interacting zones. To address these issues, several variations of the 3C method have been developed. The circular 3C, referred to as 4C, allows screening of the entire genome for sequences in contact with a specific DNA or ‘‘bait' region through inverse PCR with bait primers from a circular intermediate of 3C. However, 4C is also limited because of (1) the inability to identify interactions around the ‘bait' region; (2) the lack of resolution (~100 kb to 1 Mb); (3) the preclusion of certain interactions because of the enzymes used; and (4) the needs for validation of *trans*-interactions (interchromosomal) and distal *cis*-interactions (>500 kb from the bait) by independent methods such as fluorescence *in situ* hybridization. Another ‘upgrade' for 3C is the carbon-copy 3C (also known as 5C) that detects all chromatin interactions across large genomic regions using multiplex PCR in combination with high-throughput sequencing or microarrays.^125^ Hi-C, a method that could comprehensively detect chromatin interactions in the mammalian nucleus, was also developed.^126^ In Hi-C, a biotin-labeled nucleotide is incorporated at the ligation junction to enable selective purification of chimeric DNA ligation junctions that is then followed by deep sequencing. The compatibility of Hi-C with next-generation sequencing platforms makes it ideal to detect chromatin interactions on an unprecedented scale. Therefore, Hi-C has the power to explore both biophysical properties and structure of chromatins.^126^ ChIA-PET (chromatin interaction analysis by paired-end tag sequencing), another variation of 3C originally developed by Fullwood *et al.*^127^ to map chromatin interactions bound by estrogen receptor from breast cancer cells treated with estrogen, detects chromatin interactions bound by a defined protein. Several ChIA-PET data sets for CTCF (CCCTC-binding factor), RNA polymerase II and H3K4me2 (a chromatin modification associated with enhancers) are now available in various cell lines.^128, 129, 130^ However, as a given TF is likely only involved in a subset of chromatin interactions, ChIA-PET data sets do not include all promoter–enhancer interactions. To identify the majority of promoter–enhancer interactions, it is still necessary to use antibodies against the general TFs (such as RNA polymerase II) or chromatin modifications (such as H3K4me1 and H3K4me2) together with deep sequencing.^128, 129^

These variations of 3C techniques have already been applied in research of several common diseases except for psychiatric disorders. Using the 4C method, Patel *et al.*^131^ showed that aberrant *TAL1* expression in human T-cell acute lymphoblastic leukemia was mediated by a T-cell acute lymphoblastic leukemia-specific interchromosomal interaction between the *TAL1* promoter on chromosome 1 and a regulatory element called *TIL16* on chromosome 16.^131^ Using coined 3C with DNA selection and ligation, a 5C similar technique, Harismendy *et al.*^132^ showed that *cis*-regulatory variants associated with coronary artery disease interacted with *IFNA21*, located more than 900 kb away. The coronary artery disease risk alleles also disrupted a binding site for *STAT1*, a well-known effector of interferon signaling. This was confirmed in their following study that treatment of cells with interferon-γ increased the frequency of interaction between the enhancers and *IFNA21*.^132^ These studies have shown the great potential of chromatin-interaction approaches. Given that *cis*-regulatory elements are often highly tissue specific, future chromatin-interaction profiles generated in neuronal cell lines and brain tissues will be an invaluable resource for psychiatric studies.

Once the target gene(s) of a regulatory element has been identified using the 3C and similar technology, the impact of variant(s) on the transactivation of a specific promoter can be tested via standard reporter assay. When the regulatory potential of a certain variant is limited, such assays could map DNA regions harboring regulatory activities and provide hints for its function. Basically, regulatory elements with different alleles of the candidate variants are cloned into a promoter-driven reporter construct (for example, pGL3) and transiently transfected into relevant cell lines. This assay can be used to test the regulatory effects of variants (or haplotypes) located in either the promoter or enhancer regions. Importantly, the effect of the variant(s) might vary depending on the promoter used for reporter expression. The choice of cell type is also critical considering the high tissue- and cell-type specificity of *cis*-regulatory elements. A recent study compared variable activities of 11 enhancers across 4 mammary epithelial cell lines, and emphasized the importance of choosing appropriate cellular contexts.^133^

As the majority of regulatory functions are mediated by TFs and similar proteins, another important direction for functional analysis is to assess allele-specific protein binding. Computational prediction of TF binding based on position weight matrices models has been widely used to identify candidate TFs. With this method, quantitative scores are generated for the likelihood of observing a particular nucleotide at a specific position of the candidate TF-binding site. Recent mapping of TFs with DNA by means of ChIP-Seq provides a complementary approach that depicts the genome-wide ‘footprints' created by TFs bound to the DNA at greater sequencing depths. ChIP-Seq can also predict the regulatory status of genomic regions by targeting characteristic histone modifications. For example, promoters and enhancers are typically marked by histone methylations H3K4me3 and H3K4me1, with the additional histone acetylation mark H3K27ac indicating activation and the histone methylation mark H3K27me3 indicating repression. This approach is relatively mature and established, but several limitations still call for cautions during experimental design. First, ChIP assays do not profile more than one TF in each experiment, and the assay resolution is too low to determine the precise binding site. In addition, immunoreactivity assays such as ChIP are potentially compromised in post-mortem human brain because of the impact of post-mortem state (such as post-mortem interval and tissue pH) on epitope fidelity.^134^ Furthermore, the assay efficiency is highly dependent on the quality of the antibodies used. As a result, ChIP-Seq assay results should be interpreted considering these problems.

Electrophoretic mobility shift assays (EMSAs) can also assess protein binding *in vitro*, especially the SuperShift EMSA that determines the protein mediating allele-specific binding using antibodies against TFs of interest. In fact, several psychiatric risk loci have been analyzed with the EMSA assays for their impact on TF-binding affinity, such as *ZNF804A*,^135, 136^ *MIR137* (ref. 119) and *GRM3* (ref. 137) (Table 3). Besides, other high-throughput TF-binding methods such as proteome-wide analysis of SNPs (PWAS) using quantitative mass spectroscopy have also been used to screen SNPs for differential TF-binding affinity.^138^ This technique is very efficient as multiple SNPs and TFs can be analyzed in one experiment. For example, Butter *et al.*^138^ applied PWAS to 12 SNPs at the *IL2RA* locus associated with type 1 diabetes and narrowed down the targets to 4 SNPs showing preferential binding of common TFs. However, the *in vitro* nature of EMSAs and PWAS gives rise to false positive results, calling for verification by ChIP experiments.

In addition to tests for promoter and enhancer regulatory effects, experimental approaches for alternative splicing have also provided information for the regulatory mechanisms at psychiatric risk loci. One example is the RNA-sequencing analysis using junction data (such as alternative splicing of *AS3MT* in schizophrenia^51^) followed by functional verification with *in vitro* minigene assays. Specifically, a minigene is an artificial gene fragment containing exon(s) and necessary control regions allowing its expression in artificial conditions. The minigene has been used as a splice reporter vector (or exon-trapping vector) to determine the important factors in alternative splicing^139^ both *in vivo* and *in vitro*.^140^ Using this assay, Cohen *et al.*^141^ identified the regulatory mechanism of a schizophrenia risk SNP rs1076560 within *DRD2* in Han Chinese (Table 3). The risk SNP was associated with lower D2 short isoform expression in post-mortem brain. Further studies showed that rs1076560 abolished the ability of *ZRANB2* to modulate short versus long isoform expression ratios of *DRD2* minigene in cultured HEK293 cells, probably by disrupting a binding site for the splicing factor *ZRANB2* to diminish binding affinity between *DRD2* precursor mRNA and *ZRANB2*. In another study of *PCLO* gene (Table 3), Seo *et al.*^142^ performed functional minigene analysis of splicing regulatory sequences to characterize the function of rs13438494, a variant in the intron 24 of *PCLO* that is associated with bipolar disorder in a meta-analysis of GWAS data sets.^76^ They found that the C allele of rs13438494 reduced the splicing efficiency of the *PCLO* minigene containing exon 24, intron 24 and exon 25. In addition, prediction analysis using the Human Splice Finder web tool indicated that rs13438494 induced abrogation or creation of enhancer/silencer-binding motifs in this gene. Taken together, rs13438494 altered splicing efficiency by creating or disrupting a splicing motif and associated binding of splicing regulatory proteins.^142^ Overall, this step provides information on the regulatory mechanism of specific risk loci that usually lead to altered risk gene expression, generation of *de novo* transcripts or imbalance of the current isoform(s) expression. Such gene expression and/or processing changes are the functional parameters that can be further verified *in vitro* and *in vivo*.

## Functional analyses of genetic risk *in vitro* and *in vivo*

### Genes/isoforms

**Figure 2:**
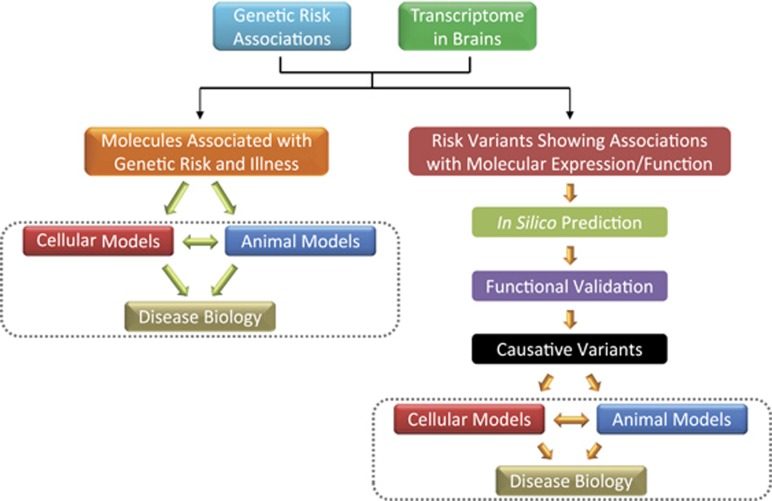
Roadmap to understand the biology of psychiatric disorders from noncoding risk loci.

Once the risk genes/transcripts associated with both genetic risk and illness state are located, genetic manipulation of their expression in cultured cells (such as rodent primary cultural neurons) and/or model animals could reveal their influence on neuronal development, brain circuit and disease-related social behaviors (Figure 2). For example, Huffaker *et al.*^49^ previously identified a primate-specific isoform (3.1) of the ether-a-go-go related K+ channel (*KCNH2*) that modulated neuronal firing to be significantly associated with schizophrenia. This risk *KCNH2*-3.1 transcript was primate specific, brain enriched and highly expressed in patients' hippocampus, whereas the canonical isoform *KCNH2*-1A was conserved between species, abundant in heart and showed no expression differences between cases and controls. Moreover, rodent primary cortical neurons overexpressed *KCNH2*-3.1 showed a rapidly deactivating K+ current and a high-frequency, nonadapting firing pattern,^49^ suggesting a novel function of this specific truncated transcript compared with *KCNH2*-1A. Later, the same group analyzed the role of *KCNH2*-3.1 using transgenic mice model, and found that mice overexpressing *KCNH2*-3.1 had significant alterations in neuronal structure and microcircuit function in the hippocampus and prefrontal cortex. These mice exhibited significant deficits in a hippocampal-dependent object location task and a prefrontal cortex-dependent T-maze working memory task.^143^ These data further strengthened the contention that *KCNH2*-3.1 was a risk factor for schizophrenia, and provided information of the neuronal basis of the disease. In recent years, there have also been studies utilizing non-human primates (such as monkeys) to characterize the pathogenic mechanisms of psychiatric risk genes. For example, Liu *et al.*^144^ reported that lentivirus-based transgenic cynomolgus monkeys (*Macaca fascicularis*) expressing human *MeCP2* in the brain showed germline transmission of the transgene and exhibited autism-like behaviors. Specifically, these *MeCP2* transgenic monkeys had a higher frequency of repetitive circular locomotion and increased stress responses compared with wild-type monkeys. The transgenic monkeys were also socially less interactive, and had a reduced interaction time when paired with other transgenic monkeys in such behavioral tests.

### Functional variants

When the causative variants of disease is identified, delving into its functional mechanism is a direct and effective approach for verification of the genetic discovery (Figure 2). The most widely used research models are *in vitro* systems such as human iPSCs and *in vivo* models with animals. Ideally, it is recommended to create panels of genetically matched iPSCs from patient somatic cells to recapitulate both normal and pathologic human tissue and organ development. These cells then serve as isogenic systems to understand disease mechanisms and to guide drug discovery with higher predictability of prognostic effects.

To study the disease-associated loci for psychiatric disorders with such systems, the common protocol is to generate isogenic human iPSC-derived neurons that differ only at the target SNP locus. This goal is usually achieved using genome-editing systems including designer endonuclease technologies such as zinc-finger nuclease, transcription activator-like effector nuclease (TALEN) and clustered regulatory interspaced short palindromic repeat (CRISPR)/Cas9 endonuclease.^145^ The consequences of these risk variants on DNA-binding protein occupancy, epigenetic modifications and gene expression in the context of neurodevelopment are then assessed. At present, many *in vitro* and *in vivo* studies involving genome-editing tools primarily focused on missense mutations. Sudhof and colleagues^146^ recently generated two different heterozygous conditional *NRXN1* mutations in human embryonic stem cells. *NRXN1* encodes neurexin-1, a presynaptic cell adhesion molecule. They found that both heterozygous *NRXN1* mutations impaired neurotransmitter release, but had no effect on synapse formation. In another study using TALENs and CRISPR/Cas9, Young-Pearse and colleagues^147^ disrupted *DISC1* near the site of the chromosome translocation found in the Scottish pedigree and found increased WNT signaling in iPSC-derived neural progenitor cells, suggesting its roles in the development of psychiatric disorders. Such studies have also provided implications for future studies on noncoding variations. As an ideal system for studying risk variants in the cellular level, human iPSCs are currently very popular in neuroscience research. However, limited access to clinical samples and difficulties in culturing and manipulating the iPSCs remain the major challenges impeding the establishment of these models.

In addition to the human iPSC systems, researchers also attempted to use model organisms (for example, mouse) to evaluate the functional consequences of the casual variants. The mouse is usually the choice of *in vivo* mammalian models for its high genome similarity, easy manipulation of genetic background and great capacity to mimic human multifactorial disease phenotypes including neural circuits and social behavior. For example, *catechol-O-methyltransferase* (*COMT*) modulates dopamine levels in the prefrontal cortex.^148^ The human *COMT* gene contains a polymorphism (Val158Met) that alters its enzyme activity and influences prefrontal cortex function,^149, 150^ and the Met allele appears to be human specific.^151, 152^ Recently, Barkus *et al.*^153^ introduced the human Met allele into the native mouse *COMT* gene to produce *COMT*-Met mice, and developed a mouse model of altered *COMT* activity comparing with their wild-type littermates. *COMT*-Met mice had reductions in *COMT* abundance and activity compared with wild-type controls. When administered with the *COMT* inhibitor tolcapoe, the attentional performance (assessed with 5-choice serial reaction time task) was only improved in wild-type mice but not in the *COMT*-Met mice.^153^ This genetic mutation knock-in mouse model provided an interesting template to study the functions of human risk variant in animals.

Though model animals provide precious information about the physiological impact of a defined genetic variant, this technique should only be used for disease-coding variants occurred in genomic regions that are highly conserved between species and have been (at best) validated using iPSCs. Comparisons between the results from human iPSCs and mouse models will demonstrate the extent to which disease variants converge on common molecular and cellular mechanisms. Meanwhile, cautions should be taken regarding several concerns in translating observations in mice to humans. For example, differences between species often exist in gene function, evolutionary conservation of genome, host responses to environmental changes and genetic backgrounds. In addition, many noncoding variations are not conserved through evolution and could only be studied *in vivo* using humanized mice. To address these concerns, simultaneous mapping of the genetic variants that already exist and introduction of new genetic variations to the model to ensure proper recapitulation of human genetic landscape are needed in future studies.

## Conclusions

Large-scale genetic studies have been successful in identifying multiple genomic loci conferring risk for psychiatric disorders. However, significant obstacles have hampered our ability to pinpoint casual variants, to identify genes/isoforms affected by causal variants and to disentangle the mechanism by which genotype influences phenotype. As opposed to linking rare mutations to Mendelian diseases, revealing the effect of common variants is a tough mission. This review provides a functional pipeline for the identification of candidate causal variants and underlying molecular mechanisms among the noncoding genetic risk loci. Unraveling the complex mechanisms underlying risk associations will ultimately pick up important biological pathways presenting suitable targets for drug development and/or reposition of known therapeutics. Steps toward filling this knowledge gap, as described in this review, will bring us closer to elucidating the genetic bases of psychiatric disorders and offer opportunities for personalized medicine.

## Figures and Tables

**Table 1:** eQTL studies in human brain^a^

*Study*	*No. of individuals*	*Tissue source*	*No. of tissue samples*	*Neuropathology*	*Age*	*Gender*	*Ethnicity*	*Expression detection methods*
Gibbs *et al.*^34^ (part of GTEx pilot)	150	Caudal pons	142	Neurologically normal controls	15–101; mean 46.2	69% Male and 31% female	Caucasian	Microarray (Illumina Human Ref-8 Expression)
		Cerebellum	143					
		frontal cortex	143					
		Temporal cortex	144					
Ramasamy *et al.*^38^ (UKBEC)	134	Occipital cortex	129	Neurologically normal controls	16–102; mean 59	74.5% Male and 25.5% female	Caucasian	Microarray (Affymetrix Huamn ST 1.0)
		Frontal cortex	127					
		Temporal cortex	119					
		Hippocampus	122					
		Intralobular white matter	131					
		Cerebellar cortex	130					
		Thalamus	124					
		Putamen	129					
		Substantia nigra	101					
		Medulla	119					
Colantuoni *et al.*^32^ (BrainCloud)	269	Dorsolateral Pre frontal cortex	269	Neurological normal controls	Fetal 80 mean 27.8	66% Male and 34% female	147 African-American; 112 Caucasian; 6 Hispanic; 4 Asian	Microarray (Illumina Human 49K Oligo array)
Liu *et al.*^41^	127	Prefrontal cortex	127	39 Bipolar disorder; 37 schizophrenia; 11 major depression; 40 controls	20–65; median 45	65% Male and 35% female	Caucasian	Microarray (Affymetrix Human Genome U133A)
Myers *et al.*^42^	193	Cortex (pooled data from 20% frontal, 70% temporal and 1% parietal)	193	Neurological normal controls	65–100; average 81	54% Male and 46% female	Caucasian	Microarray (Illumina Human Refseq-8)
Webster *et al.*^43^	364	Cortex (pooled from 21% frontal, 73% temporal, 2% parietal and 3% cerebellar)	188	Neurological normal controls	65–100; average 81	55% Male and 45% female	Caucasian	Microarray (Illumina Human Refseq-8)
		Cortex (pooled from 18% frontal, 60% temporal, 10% parietal and 13% cerebellar)	176	Patients with late-onset Alzheimer's disease	68–102; average 84	50% Male and 50% female	Caucasian	
Heinzen *et al.* ^40^ (SNPExpress)	93	Frontal cortex	93	Neurological normal controls	34–90; mean 74	59% Male and 41% female	Caucasian	Microarray (Affymetrix Huamn ST 1.0)
Zou *et al.* ^44^	~400	Cerebellum	197	Patients with Alzheimer's disease	Mean±s.d.; 73.6±5.6	49% Male and 51% female	Caucasian	Microarray (Illumina HumanHT−12 v4.0)
			177	Patients with other brain pathologies	Mean ± s.d.; 71.7 ± 5.5	64% Male and 36% female	Caucasian	
		Temporal cortex	202	Patients with Alzheimer's disease	Mean±s.d.; 73.6±5.5	47% Male and 53% female	Caucasian	
			197	Patients with other brain pathologies	Mean±s.d.; 71.6±5.6	60% Male and 40% female	Caucasian	
GTEx, v6^35, 36^	72–103	Anterior cingulate caudate, caudate (basal ganglia), cerebellar hemisphere, cerebellum, cortex, frontal cortex, hippocampus, hypothalamus, nucleus accumbens (basal ganglia), putamen (basal ganglia)	NA	Neurological normal controls	NA	NA	NA	RNA-sequencing (polyA)
UKBEC (unpublished)	65–105	Substantia nigra, putamen	NA	Neurological normal controls	NA	NA	Caucasian	RNA-sequencing
Lieber Institute (unpublished)	>700	Dorsolateral prefrontal cortex, hippocampus	NA	Patients with bipolar disorder, schizophrenia and major depressive disorder, and neurological normal controls	NA	NA	NA	RNA-sequencing
CommonMind consortium^39^	537	Dorsolateral prefrontal cortex	537	258 Patients with schizophrenia; 279 controls	NA	NA	Caucasian 80.7% African-American 14.7% Hispanic 7.7% East Asian 0.6%	RNA-sequencing (RiboZero)

Abbreviations: GTEx, Genotype-Tissue Expression; eQTL, expression quantitative trait locus; NA, not available; UKBEC, United Kingdom Brain Expression Consortium.

Websites: GTEx http://www.gtexportal.org/home/.

UKBEC http://www.braineac.org/.

BrainCloud http://braincloud.jhmi.edu/BrainCloud64/BrainCloud64bit.htm.

SNPExpress http://igm.cumc.columbia.edu/SNPExpress/.

CommonMind http://commonmind.org/WP/.

a Nonexhaustive list of examples.

**Table 2:** Computational tools and resources for the analyses of noncoding risk loci^a^

*Feature*	*Description*	*Significance*	*Experimental approach*	*Bioinformatic tools and online resources*
Open chromatin	Nucleosome-depleted chromatin	DNA sequences harboring regulatory signals	DNase-seq, FAIRE sequencing	ENCODE,^96^ REMC,^97^ RegulomeDB,^98^ HaploReg,^99^ FunciSNP^101^
TF-binding prediction	Short DNA consensus recognition sequence characteristic of a particular DNA-binding protein	Computationally predicted TF recognition site	Position weight matrices	TRANSFAC,^154^ JASPAR,^155^ MAPPER2,^156^ GWAS3D,^157^ DeepSEA^158^
DNA–protein interaction	Short DNA sequence associated with a DNA-binding protein after precipitation with a specific antibody	Physical protein-nucleic-acid binding	ChIP-seq, DNase footprinting	ENCODE,^96^ NRCistrome,^159^ RegulomeDB,^98^ HaploReg,^99^ GWAVA^100^
DNA methylation	Methylation of cytosine residues in CpG dinucleotides	Regulation of gene expression	Methylation array, bisulfite sequencing, MeDIP-seq, MRE-seq	ENCODE,^96^ REMC,^97^ MethDB,^160, 161^ EpiGraph,^162^ BrainCloudMethyl,^163^ Fetal brain meQTLs^107^
DNase I hypersensitive sites	Sensitive to cleavage by the DNase I enzyme	DNA sequences harboring regulatory signals	DNase-seq	ENCODE,^96^ REMC,^97^ PsychENCODE,^113^ DeepSEA^158^
Histone modifications	Specific posttranslational modifications of particular histone protein residues are associated with various regulatory activities	H3K4me1: promoters and enhancers H3K4me3: promoters H3K27ac: active regulatory region H3K9ac: promoters H3K9me1: active chromatin	ChIP-seq	ENCODE,^96^ REMC,^97^ PsychENCODE,^113^ NRCistrome,^159^ RegulomeDB,^98^ HaploReg,^99^ ChromHMM,^164^ GWAS3D,^157^ ChroMoS,^165^ SEA,^166^ DeepSEA^158^
Chromatin interactions	Long-range physical interactions between distal genomic regions	Contact between regulatory motifs, such as tissue-specific enhancers and promoters	3C, 4C, 5C, Hi-C, ChIA-PET	GWAS3D,^157^ Hi-C Browser,^167^ CCSI^168^
MicroRNA-binding prediction	Short DNA consensus recognition sequence characteristic of a particular microRNA	Computationally predicted microRNA recognition site	Position weight matrices	miRanda,^169^ Target Scan,^170^ MicroSNiPer^171^

Abbreviations: 3C, chromosome conformation capture; 4C, circular 3C; 5C, carbon-copy 3C; CCSI, Chromatin Chromatin Space Interaction; ChIA-PET, chromatin interaction analysis by paired-end tag sequencing; ChIP-Seq, chromatin immunoprecipitation followed by next-generation sequencing; DNase-seq, DNase I hypersensitive site sequencing; FAIRE, formaldehyde-assisted isolation of regulatory elements; MeDIP-seq, methylated DNA immunoprecipitation sequencing; MRE-seq, methylation-sensitive restriction enzyme sequencing; NRCistrome, Nuclear Receptor Cistrome; REMC, NIH Roadmap Epigenomics Project; RNA-PET, RNA paired-end tag sequencing; SEA, super-enhancer archive; TF, transcription factor.

Websites: ENCODE https://www.encodeproject.org/.

REMC http://www.roadmapepigenomics.org/.

RegulomeDB http://www.regulomedb.org.

HaploReg http://www.broadinstitute.org/mammals/haploreg.

FunciSNP http://bioconductor.org/packages/2.12/bioc/html/FunciSNP.html.

TRANSFAC http://www.gene-regulation.com/index2.

MAPPER2 http://genome.ufl.edu/mapperdb.

GWAS3D http://jjwanglab.org/gwas3d/.

DeepSEA http://deepsea.princeton.edu/job/analysis/create/.

NRCistrome http://www.cistrome.org/Cistrome/Cistrome_Project.html.

GWAVA http://www.sanger.ac.uk/sanger/StatGen_Gwava.

MethDB http://www.methdb.de.

EpiGRAPH http://epigraph.mpi-inf.mpg.de/WebGRAPH/.

BrainCloudMethyl http://braincloud.jhmi.edu/Methylation64/BrainCloudMethyl64bit.htm.

Fetal brain meQTLs http://epigenetics.essex.ac.uk/mQTL/.

PsychENCODE http://psychencode.org/.

ChromHMM http://compbio.mit.edu/ChromHMM/.

ChroMoS http://epicenter.immunbio.mpg.de/services/chromos.

SEA http://www.bio-bigdata.com/SEA/.

Hi-C Browser http://hic.umassmed.edu/welcome/welcome.php.

CCSI http://songyanglab.sysu.edu.cn/ccsi/search.php.

miRanda http://www.microrna.org/microrna/home.do.

Target Scan http://www.targetscan.org/vert_71/.

MicroSNiPer http://epicenter.ie-freiburg.mpg.de/services/microsniper/.

a Nonexhaustive list of examples.

**Table 3:** Functional genetic variants successfully identified at psychiatric risk loci^a^

*Disease or phenotype*	*Locus*	*Functional variants*	*Target genes*	*Key methods*	*References*
Schizophrenia, bipolar disorder	1p21.3	1:g.98515539A>T	*MIR137/MIR2682*	3C, EMSA, reporter assays	^119^
Schizophrenia, bipolar disorder	2q32.1	rs1344706	*ZNF804A*	eQTL, EMSA,	^52, 136^
Schizophrenia	2q32.1	rs359895	*ZNF804A*	EMSA, reporter assays	^135^
Bipolar disorder	7q21.11	rs13438494	*PCLO*	splicing assays	^142^
Bipolar disorder	7q21.1–q21.2	rs148754219	*GRM3*	eQTL, EMSA, reporter assays	^137^
Schizophrenia	10q24.32	VNTR	*AS3MT*	eQTL, reporter assays	^51^
Schizophrenia	11q23	rs1076560	*DRD2*	eQTL, splicing assays	^141^
Schizophrenia	12p13.3	rs2159100/rs12315711	*CACNA1C*	3C, reporter assays	^120^
Schizophrenia	12p13.3	rs1006737/rs4765905	*CACNA1C*	eQTL, 4C, reporter assays, protein arrays	^124^

Abbreviations: 3C, chromosome conformation capture; 4C, circular 3C; EMSA, electrophoretic mobility shift assay; eQTL, expression quantitative trait locus.

a Nonexhaustive list of examples. It should be noted that some of these genetic loci are positive only in candidate gene studies but not in genome-wide association studies (GWASs).
